# Differential Semiology in Video-EEG Monitoring: A Clinical Approach to Distinguishing Psychogenic Nonepileptic and Epileptic Seizures

**DOI:** 10.7759/cureus.108782

**Published:** 2026-05-13

**Authors:** Natasa Pejanovic-Skobic, Nikola Krtalic, Marija Bender, Nikolina Pravdic, Davor Batinic

**Affiliations:** 1 Clinic of Neurology, University Clinical Hospital Mostar, Mostar, BIH; 2 School of Medicine, University of Mostar, Mostar, BIH; 3 Child Health Services, Health Center Mostar, Mostar, BIH

**Keywords:** diagnostic accuracy, differential diagnosis, epilepsy, psychogenic nonepileptic seizures, seizure semiology, video-eeg monitoring

## Abstract

Introduction

Distinguishing psychogenic nonepileptic seizures (PNES) from epileptic seizures (ES) remains a major diagnostic challenge in clinical neurology. Misdiagnosis is common and may result in inappropriate treatment, including unnecessary use of antiepileptic drugs and delayed psychiatric care. Video-electroencephalographic (video-EEG) monitoring is considered the gold standard for diagnosis, but it is not universally available. Semiological features, therefore, remain an essential diagnostic tool, particularly in resource-limited settings.

Methods

This retrospective observational study included 43 patients who underwent video-EEG monitoring at the Clinic of Neurology, University Clinical Hospital Mostar, between May 2022 and May 2025. Patients were classified into ES and PNES groups based on video-EEG findings. Multiple clinical and semiological features were analyzed. Statistical significance was defined as p < 0.05.

Results

PNES were significantly associated with seizure duration >2 minutes (71.4% vs. 10.3%, p < 0.001), gradual onset, preserved responsiveness, eye closure with resistance to passive opening, and fluctuating course. ES were characterized by sudden onset, shorter duration, impaired responsiveness, and head deviation. The combination of these semiological features showed clear discriminatory value in differentiating PNES from ES.

Conclusion

Semiological analysis represents a valuable and accessible diagnostic tool in differentiating PNES from ES. Its systematic application may improve early diagnostic accuracy, reduce diagnostic delay, and optimize patient management, particularly in settings with limited access to video-EEG monitoring.

## Introduction

Epileptic seizures (ES) are defined as transient occurrences of signs or symptoms resulting from abnormal, excessive, or synchronous neuronal activity in the brain [1]. Epilepsy, as a chronic neurological disorder, is characterized by a persistent predisposition to generate such seizures and is associated with a wide spectrum of neurobiological, cognitive, psychological, and social consequences [2,3].

Functional seizures, previously termed psychogenic nonepileptic seizures (PNES), are paroxysmal events that resemble ES but are not associated with epileptiform EEG activity [4,5]. They are generally classified within the spectrum of functional neurological or dissociative disorders [6] and represent one of the most frequent differential diagnoses in epilepsy practice [7].

The distinction between PNES and ES remains a major clinical challenge. Misdiagnosis is common, with studies suggesting that a substantial proportion of patients referred for pharmacoresistant epilepsy ultimately receive a diagnosis of PNES [7]. Such diagnostic errors may lead to prolonged and unnecessary treatment with antiseizure medications, increased healthcare costs, and delayed access to appropriate psychological or psychiatric care [8,9]. Furthermore, diagnostic delay in PNES can extend over several years, negatively affecting patient outcomes and quality of life [10].

Video-electroencephalographic (video-EEG) monitoring is widely regarded as the gold standard for distinguishing PNES from ES, as it enables simultaneous recording of clinical behavior and EEG activity [11]. However, access to this modality is often limited due to cost, technical requirements, and availability of specialized centers [12]. Consequently, clinicians frequently rely on detailed clinical assessment and seizure semiology as essential diagnostic tools [13].

Semiology, defined as the systematic analysis of clinical signs and symptoms during seizures, provides valuable information regarding the origin and nature of paroxysmal events [14,15]. Numerous studies have identified specific semiological features that may help differentiate PNES from ES, including seizure duration, mode of onset, responsiveness, eye closure, and motor patterns [16,17]. Nevertheless, no single feature is entirely specific, and diagnostic accuracy typically depends on the combined interpretation of multiple signs [17].

The aim of this study was to identify key semiological differences between PNES and ES in patients undergoing video-EEG monitoring and to evaluate their potential clinical utility in improving diagnostic accuracy. Although several studies have evaluated semiological differences between ES and functional seizures, data from smaller single-center video-EEG cohorts remain clinically valuable, particularly in reflecting real-world diagnostic practice in developing epilepsy monitoring units. The present study aimed not only to identify distinguishing semiological features within our cohort but also to assess whether our findings are consistent with previously reported observations in the literature.

## Materials and methods

This retrospective observational study was conducted at the video-EEG monitoring unit of the Clinic of Neurology, University Clinical Hospital Mostar, between May 1, 2022, and May 1, 2025. The study setting represents a tertiary referral center where patients with suspected epilepsy, pharmacoresistant seizures, or unclear paroxysmal events are routinely evaluated using prolonged video-EEG monitoring [18].

A total of 43 patients aged 18 to 65 years were included in the study. All patients experienced at least one recorded event during video-EEG monitoring, enabling definitive classification. Indications for referral included diagnostic uncertainty, suspected nonepileptic events, pharmacoresistant epilepsy, and presurgical evaluation [18].

Diagnosis of ES or PNES was established based on the correlation between clinical semiology and EEG findings. PNES were diagnosed when typical clinical events occurred without corresponding epileptiform EEG activity, in accordance with established criteria [19,20].

Semiological analysis was performed through systematic review of recorded events and included a detailed assessment of seizure characteristics such as duration, onset type, occurrence during sleep or wakefulness, eye closure with resistance to passive opening, head movements, pelvic thrusting, voluntary limb movements, responsiveness, fluctuation of seizure course, and vocalization patterns [14,16].

All video-EEG recordings were reviewed by experienced clinicians trained in epilepsy diagnostics. Informed consent was obtained from all patients. Continuous monitoring was performed using standardized protocols, enabling precise electroclinical correlation.

Statistical analysis was performed using IBM SPSS Statistics for Windows, Version 25 (Released 2017; IBM Corp., Armonk, New York, United States). Continuous variables were expressed as median with interquartile range or mean ± standard deviation, as appropriate. Age was compared between groups using the Mann-Whitney U test. Comparisons between groups were performed using the chi-square test or Fisher’s exact test, as appropriate. The chi-square test was used for variables with sufficient expected frequencies, including seizure duration, gradual onset, eye closure, preserved responsiveness, fluctuating course, and head deviation. Fisher’s exact test was applied for variables with small cell counts, including pelvic thrusting, voluntary limb movements, and atypical vocalization. Statistical significance was defined as p < 0.05.

The study was approved by the Ethics Committee of the School of Medicine, University of Mostar (no: 01-1-401/25), and conducted in accordance with the ethical principles outlined in the Declaration of Helsinki.

## Results

Demographic characteristics

A total of 43 patients were included in the study, all of whom were admitted and evaluated at the video-EEG monitoring unit of the Clinic of Neurology, University Clinical Hospital Mostar, between May 1, 2022, and May 1, 2025. Among them, 29 patients (67.4%) were diagnosed with ES, while 14 patients (32.6%) were diagnosed with PNES. This distribution was statistically significant (χ^2^ = 5.233, df = 1, p = 0.022).

The median age of the cohort was 29 years (IQR 21-39; range 18-65 years). There was no statistically significant difference in age between groups (Mann-Whitney U = 140, p = 0.10). There was also no statistically significant difference in sex distribution between the ES and PNES groups (χ^2^ = 0.433, df = 1, p = 0.720) (Table 1).

**Table 1:** Demographic characteristics of patients with ES and PNES ^1^ Mann-Whitney U test (U = 140, two-tailed p = 0.10). ES: epileptic seizures; PNES: psychogenic nonepileptic seizures

Variable	ES (n = 29), n (%)	PNES (n = 14), n (%)	p-value
Age (years), median (IQR)	29 (21-39)	29 (21-39)	0.10^1^
Male	9 (31.0)	3 (21.4)	0.720
Female	20 (69.0)	11 (78.6)	-

Core semiological features

Several key semiological features differed significantly between groups. PNES were significantly associated with longer seizure duration (>2 minutes) and gradual onset. Patients with PNES frequently demonstrated eye closure with resistance to passive opening, preserved responsiveness during the event, and a fluctuating seizure course characterized by variability in intensity and motor activity. In contrast, ES were more commonly characterized by sudden onset, shorter duration, and impaired responsiveness. Head deviation was significantly more frequent in patients with ES, suggesting focal neurological involvement (Table 2).

**Table 2:** Key semiological features distinguishing epileptic seizures (ES) and psychogenic nonepileptic seizures (PNES)

Feature	ES (n = 29), n (%)	PNES (n = 14), n (%)	p-value
Seizure duration >2 min	3 (10.3)	10 (71.4)	<0.001
Gradual onset	11 (37.9)	11 (78.6)	0.012
Eye closure with resistance	1 (3.4)	11 (78.6)	<0.001
Preserved responsiveness	3 (10.3)	11 (78.6)	<0.001
Fluctuating course	3 (10.3)	8 (57.1)	0.002
Head deviation	13 (44.8)	1 (7.1)	0.016

Significant differences were observed for seizure duration (χ^2^ = 16.702, p < 0.001), eye closure with resistance (χ^2^ = 26.485, p < 0.001), preserved responsiveness (χ^2^ = 20.016, p < 0.001), and fluctuating course (χ^2^ = 10.862, p = 0.002).

Additional semiological features

Certain features, including pelvic thrusting, voluntary limb movements, and vocalization, did not show statistically significant differences between groups, indicating that these signs alone have limited diagnostic specificity (Table 3).

**Table 3:** Additional semiological features ES: epileptic seizures; PNES: psychogenic nonepileptic seizures

Feature	ES (n = 29), n (%)	PNES (n = 14), n (%)	p-value
Pelvic thrusting	0 (0.0)	1 (7.1)	0.326
Voluntary limb movements	3 (10.3)	4 (28.6)	0.190
Atypical vocalization	2 (6.9)	4 (28.6)	0.077

These findings support the use of a structured semiological approach in the clinical evaluation of patients with suspected seizure disorders. The presence of multiple features such as prolonged duration, eye closure with resistance, preserved responsiveness, and fluctuating course should raise a strong suspicion of PNES. On the other hand, sudden onset, short duration, and impaired awareness are more consistent with ES.

These findings reinforce the role of bedside clinical assessment in early diagnostic orientation. Such an approach may be particularly valuable in settings where immediate access to video-EEG monitoring is limited, allowing clinicians to make more informed decisions regarding further diagnostic evaluation and management.

## Discussion

The present study demonstrates that several semiological features show clear and clinically meaningful differences between PNES and ES, supporting and extending previously published findings in this field [13,21,22]. Eye closure with resistance to passive opening emerged as one of the most distinctive features associated with PNES, consistent with earlier studies highlighting its high diagnostic value [23]. Similarly, preserved responsiveness and fluctuating seizure course further support the functional nature of PNES. In contrast, ES were characterized by abrupt onset, impaired consciousness, and more stereotyped clinical patterns, reflecting the underlying pathophysiology of synchronous neuronal discharge [13]. Head deviation observed in ES patients is also consistent with focal seizure semiology and neuroanatomical localization [15].

Importantly, our findings reinforce the concept that no single semiological feature is sufficient for reliable differentiation between PNES and ES. This observation is consistent with previous meta-analyses and supports a multidimensional diagnostic approach that integrates semiology, clinical history, and EEG data [17,24].

Recent literature further supports these findings. Duncan et al. emphasized the diagnostic value of fluctuating seizure course in epilepsy monitoring units [13], while Muthusamy et al. confirmed that combinations of semiological features provide the highest diagnostic accuracy [22]. Additionally, Pevy et al. highlighted the importance of detailed clinical assessment and patient narrative in differentiating epileptic and functional seizures [25].

An additional diagnostic challenge is the recognized coexistence of ES and functional seizures in some patients. Previous studies have demonstrated that both conditions may occur in the same individual, occasionally even within the same clinical episode, further complicating bedside differentiation and emphasizing the importance of prolonged video-EEG monitoring for accurate diagnosis. From a clinical perspective, early recognition of PNES can prevent unnecessary use of antiseizure medications and facilitate timely psychological intervention [26,27]. Given that diagnostic delay can extend for several years [10], improving clinical recognition of semiological patterns remains essential.

While many of the identified semiological features are familiar to epilepsy specialists, their systematic presentation remains clinically important for general neurologists and physicians working outside specialized epilepsy centers, particularly in resource-limited settings where access to prolonged video-EEG monitoring may be restricted. In such settings, careful semiological assessment can play a crucial role in reducing diagnostic delay and avoiding inappropriate treatment [13].

The study should be interpreted in light of several limitations. First, the relatively small sample size may limit the statistical power and generalizability of the findings. However, all included patients underwent video-EEG monitoring with definitive electroclinical classification, which ensures high diagnostic accuracy and strengthens the validity of the results. Second, the retrospective single-center design may introduce selection bias and limit external applicability. Nevertheless, the study reflects real-world clinical practice in a tertiary referral center and provides clinically relevant insights into routine diagnostic challenges.

Future studies should aim to include larger, multicenter cohorts and explore the development of standardized semiological scoring systems or predictive models that could further improve diagnostic accuracy. Integration of semiological features with advanced analytical approaches may represent an important direction for future research.

## Conclusions

In conclusion, this study demonstrates that structured semiological assessment represents a clinically valuable and accessible tool for differentiating PNES from ES. The combination of multiple semiological features - particularly prolonged seizure duration, eye closure with resistance to passive opening, preserved responsiveness, and fluctuating course - provides significantly greater diagnostic accuracy than any single clinical sign. These findings have important clinical implications, particularly in settings where access to video-EEG monitoring is limited. Early recognition of PNES based on semiological patterns can reduce misdiagnosis, prevent unnecessary use of antiseizure medications, and facilitate timely psychiatric and psychological intervention. Integrating structured semiological analysis into routine clinical evaluation may therefore improve diagnostic efficiency, optimize patient management, and reduce healthcare burden associated with delayed or incorrect diagnosis.
